# PATHOS: Pathology attention framework for treatment response stratification in ovarian high-grade serous carcinomas following neoadjuvant chemotherapy on H&E images

**DOI:** 10.1016/j.jpi.2026.100545

**Published:** 2026-01-21

**Authors:** Francesca Miccolis, Marta Lovino, Oskari Lehtonen, Johanna Hynninen, Sampsa Hautaniemi, Anni Virtanen, Elisa Ficarra

**Affiliations:** a DIEF, University of Modena and Reggio Emilia, Via Vivarelli, 10, Modena 41125, Italy; b Life Science Department, University of Modena and Reggio Emilia, Via Campi, 287, Modena 41121, Italy; c Research Program in Systems Oncology, Research Programs Unit, Faculty of Medicine, University of Helsinki, Fabianinkatu 33, Helsinki 00100, Finland; d Department of Pathology, University of Helsinki and HUS Diagnostic Center, Helsinki University Hospital, Haartmaninkatu 3, Helsinki 00260, Finland; e Department of Obstetrics and Gynecology, University of Turku and Turku University, Kiinamyllynkatu 10, Turku 20520, Finland

**Keywords:** Digital pathology, Explainable AI (XAI), Ovarian high-grade serous carcinoma (HGSC), Multiple instance learning (MIL), Neoadjuvant chemotherapy (NACT), Panoptic segmentation, Treatment response prediction

## Abstract

Ovarian high-grade serous carcinoma (ovarian HGSC) is a clinically challenging disease with a poor prognosis, particularly for patients receiving neoadjuvant chemotherapy (NACT) before debulking surgery. In this study, we evaluate the progression-free interval (PFI) after NACT based on hematoxylin and eosin-stained whole-slide images (WSIs) of omental tumor tissue. Digital pathology tools are emerging, aiming at assisting pathologists in diagnosis and analysis; however, distinguishing features associated with response to NACT remain elusive. Multiple instance learning (MIL) coupled with attention mechanisms has shown promise in predicting treatment response from WSIs. Additionally, segmentation tools can identify and delineate regions in WSIs. Whereas some efforts have been made to develop explainable models for clinical outcome, there remains a need for genuinely interpretable models for pathologists. This article introduces the PATHOS framework, a novel approach to explaining crucial features of treatment response based on the PFI time in NACT treated patients from WSIs. PATHOS is composed of three blocks: (1) MIL block to identify informative regions, (2) panoptic segmentation and downstream analysis block for feature computation, and (3) classification block to predict the PFI. The results demonstrate that PATHOS enhances the interpretability of response to NACT in ovarian HGSC patients by highlighting pathologically significant features relevant to PFI prediction, such as tumor cell morphology, stromal abundance, and the spatial distribution of stromal regions. Furthermore, PATHOS identifies approximately 10% of the total WSI area as an informative region for clinical outcome.

## Highlights

• PATHOS uses MIL and segmentation to predict treatment response in ovarian HGSC.
• Identifies stromal and morphological features linked to platinum-free interval.
• Isolates a critical 10% WSI region indicative of treatment response.
• Enhances interpretability, supporting explainable AI in digital pathology.

## Introduction

Ovarian high-grade serous carcinoma (HGSC) is a highly aggressive malignancy with a poor prognosis and a complex histopathological landscape characterized by high mortality rates and intricate tumor biology.^13^^,^^15^ Patients with advanced-stage ovarian HGSC often undergo neoadjuvant chemotherapy (NACT) before debulking surgery, yet the 5-year survival rate remains disappointingly low, at approximately 25%.^16^ The variability in treatment response among patients poses a significant clinical challenge. Whereas digital pathology tools have revolutionized the analysis of whole-slide images (WSIs), extracting meaningful insights from WSIs remains challenging due to their high dimensionality and variability. This complexity is further exacerbated in the context of NACT, where treatment-induced morphological changes can be subtle and heterogeneous, making it difficult to identify reliable biomarkers for predicting treatment response. Consequently, there is an urgent need for accurate and interpretable methods to characterize the histopathological features associated with treatment response in HGSC.

Multiple instance learning (MIL)^5^^,^^25^ has emerged as a powerful tool for analyzing image data, particularly in the context of pathology. MIL models excel at handling heterogeneous data and can effectively learn representations from WSIs.^20^^,^^31^ By leveraging attention mechanisms, MIL models can potentially identify the most informative regions within an image, providing valuable insights into the underlying biological processes. However, whereas MIL models generate attention maps (heatmaps where the color code indicates relevance for prediction), the interpretation of these maps often remains challenging, hindering their clinical utility. The key strength of MIL models is their ability to leverage global-level information, such as patient or WSI labels, without requiring detailed annotations, at patch- or pixel level. This makes MIL particularly well-suited for predicting treatment response based on WSIs, as the available data are often limited to the patient- or slide level.^12^^,^^19^

State-of-the-art (SOTA) attention-based MIL approaches include DSMIL,^18^ GDS-MIL,^2^ and BufferMIL.^3^ DSMIL model selects the most critical patch of a WSI, leveraging the MIL attention mechanism to assign a weighted score for each patch to represent its contribution in the prediction of treatment response.^5^^,^^7^ GDS-MIL model leverages a graph-based dual-stream architecture to capture both local patch relevance and inter-patch spatial relationships. Whereas DSMIL takes only one patch as the critical one for the prediction, BufferMIL takes a buffer of patches to drive the prediction of the entire WSI. In conclusion, the MIL approach stands out as a robust method for the analysis of histopathological WSIs, effectively addressing the challenges posed by high-resolution images and the need for efficient annotation.

Despite its success across various medical imaging tasks, a notable gap remains in the literature regarding the interpretability of existing MIL-based models applied to ovarian cancer. Specifically, these models have yet to produce a set of features that pathologists can reliably use in practice.^22^ Therefore, whereas recent advancements in MIL algorithms offer substantial progress, the interpretability aspect remains under-explored.

Panoptic segmentation, another advanced computer vision technique, offers the potential to extract detailed spatially resolved information from WSIs by simultaneously segmenting semantic regions^14^^,^^30^ such as tissues and countable objects like cells or nuclei. Indeed, it can reveal the spatial distribution of cells within different tissue compartments and allows for the identification of subtle localized features in WSIs that enhance digital pathology workflows for interpretable feature extraction. Whereas panoptic segmentation offers detailed insights into tissue architecture, a significant limitation is the time required to segment entire WSIs, which can hinder its scalability for large-scale studies.

To overcome the limitations of both MIL models and panoptic segmentation, the strengths of both approaches are integrated, proposing a novel PATHOS framework designed to explain crucial features of treatment response in ovarian HGSC patients based on hematoxylin and eosin (H&E) WSI analysis.

The achieved results demonstrate that PATHOS offers a robust and interpretable approach to predicting treatment response in ovarian HGSC. The model integrates both MIL and panoptic segmentation, addressing the challenges of traditional approaches by providing clear explanations of pathological features associated with treatment response in ovarian HGSC patients undergoing NACT. By leveraging these techniques, PATHOS quantitatively refines and prioritizes a group of histopathological features previously associated with platinum response,^4^ whose spatial patterns and combined relevance are challenging to objectively and consistently quantify through manual evaluation—such as morphological irregularities of tumor cells, complexity of tumor cell shape, density and spatial extent of stromal tissue, and relative abundance of stromal cells, contributing to the prediction of treatment response in patients undergoing NACT treatment.

## Materials and methods

### Dataset

The dataset contains 176 H&E-stained WSIs of omental tumor obtained at the time of interval debulking surgery, following NACT. Omental tumor deposits were selected due to their high prevalence and standardized sampling in high-grade serous ovarian cancer. Moreover, prior studies have demonstrated that omental tumor morphology following NACT contains predictive information about platinum sensitivity and overall treatment outcome.^4^^,^^8^ Critically, the omentum is recognized as a key metastatic niche, where its unique microenvironment, rich in adipocytes and immune cells, actively promotes the proliferation and metastatic dissemination of HGSC cells.^9^ Furthermore, the assessment of omental metastasis is directly linked to prognostic risk factors for unfavorable outcomes in HGSC, enhancing its utility in revealing clinically relevant morphological differences.^11^ The cohort consists of 50 patients diagnosed with HGSC (DECIDER observational clinical trial—Multi-layer Data to Improve Diagnosis, Predict Therapy Resistance and Suggest Targeted Therapies in HGSC; ClinicalTrials.gov identifier: NCT04846933).

In this study, treatment response in ovarian HGSC is assessed using the PFI, defined as the time from completion of platinum-based chemotherapy to the first documented disease recurrence or progression, and considered a critical indicator of a patient's sensitivity or resistance to platinum therapy. The dataset was analyzed with 5-fold cross-validation, stratifying by patient to keep all WSIs from the same patient within the same set. The prediction task is framed as a binary classification problem based on clinically meaningful PFI^10^:

• PFI > 365 days: these patients have a good response to platinum therapy, given the extended recurrence-free interval, suggesting continued sensitivity to platinum. This class is referred to as the *long PFI*. The dataset contains 87 *long PFI* WSIs for 25 patients.
• PFI < 180 days: patients in this category have a poor response to platinum-based chemotherapy, as the short interval implies quick recurrence and likely platinum resistance. For the sake of this study, this class is referred to as the *short PFI* class. The dataset comprises 92 *short PFI* WSIs for 25 patients.

### Framework architecture

**Fig. 1:**
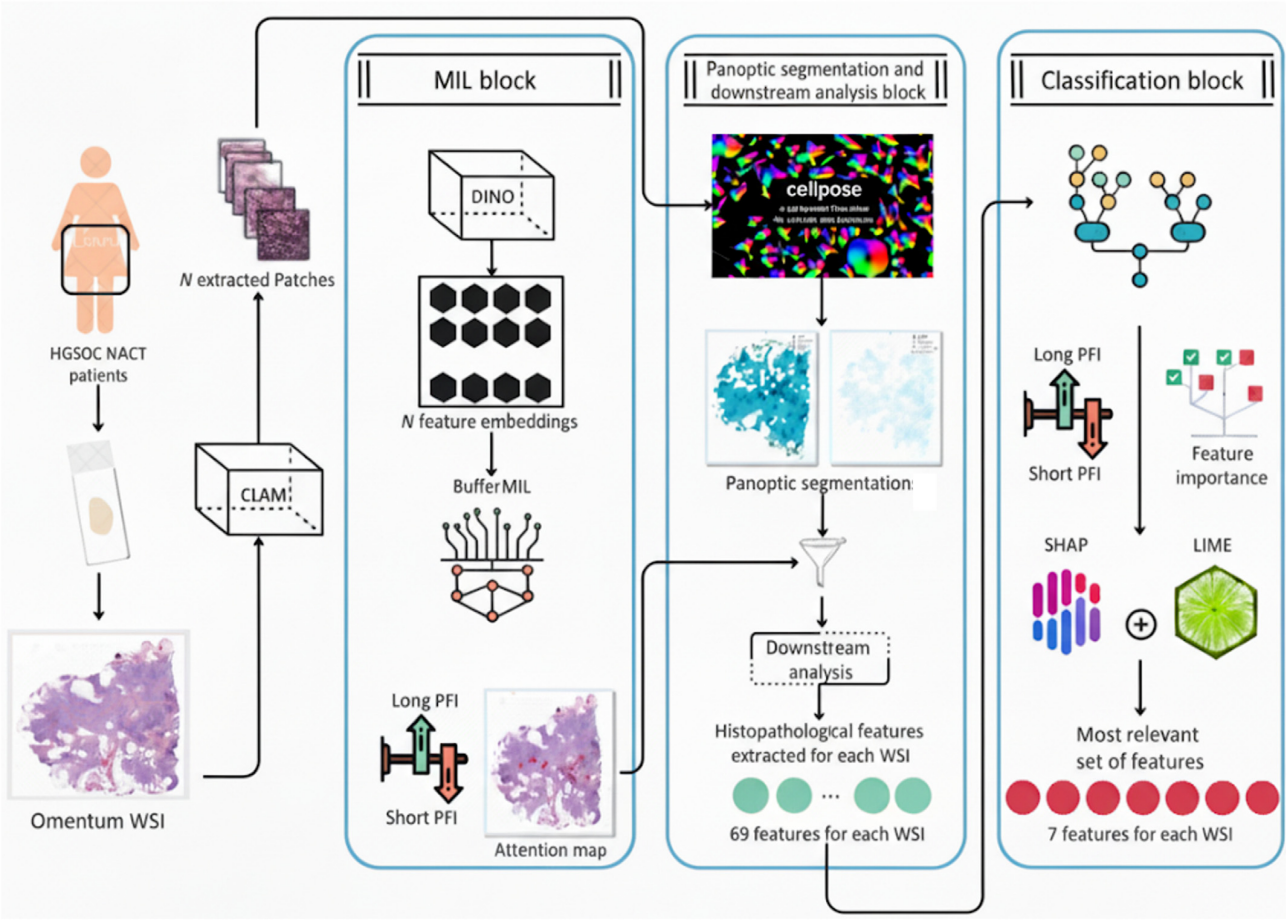
PATHOS architecture: whole-slide images from ovarian high-grade serous carcinoma treated patients are processed in three stages; (1) MIL block: patches extracted via CLAM are encoded with DINO and analyzed with BufferMIL to identify informative regions in WSIs and PFI prediction. (2) Panoptic segmentation and downstream analysis block: a fine-tuned version of the CellPose segments cells and tissue structures; the segmentation results are processed with the downstream analysis to extract 69 histopathological features for each WSI. (3) Classification block: multiple state-of-the-art machine learning classifiers were employed to predict PFI based on the extracted features. Model interpretability was ensured through SHAP and LIME, enabling the identification of feature contributions across different classifiers, ultimately resulting in the selection of the top seven most informative features for PFI prediction.

The proposed framework is represented in Fig. 1 and is composed of three elements:

1. The MIL block to identify informative regions, exploiting BufferMIL.^3^
2. The panoptic segmentation and downstream analysis block to extract histopathological features.
3. The classification block, which provides the final prediction based on the extracted histopathological features deemed significant and interpretable by pathologists.

#### MIL block

Given the histomorphological, molecular, and microenvironmental heterogeneity of ovarian HGSC, the BufferMIL model was selected.^3^ This model relies on predicting a batch of patches rather than a single one, thereby capturing the diverse and complex nature of ovarian HGSC and leading to more accurate and robust predictions. Patches are first extracted from each WSI using CLAM^24^ with default parameters. Then DINO,^6^ a self-supervised task-agnostic model, is employed to generate an unsupervised representation for each patch (384-dimensional vector), representing the histopathological information in a compact form. Consequently, each WSI is represented by *N* 384-dimensional vectors, where *N* denotes the total number of patches extracted from the WSI.

Then, BufferMIL was applied employing a 5-fold cross-validation technique with label stratification to ensure class balance in each fold. BufferMIL produces a binary prediction—*short PFI* or *long PFI*—and an attention map for each WSI. The attention map is a discretized heatmap, reporting the relative importance of each patch toward the *short/long PFI* prediction.

#### Panoptic segmentation and downstream analysis block

Panoptic segmentation, which simultaneously segments nuclei and tissue regions, was employed to enable the quantification of interpretable features in a spatially aware manner. The panoptic segmentation is implemented with the *cellseg_models.pytorch* library, employing a Cellpose-based^29^ model trained on a semimanually labeled ovarian HGSC training dataset (details are reported in Appendix Table A.1) that included six tissue region types (stroma, benign omental tissue, epithelial tumor, necrosis, hemorrhage, and serum) and five distinct nuclear types (neoplastic, connective, inflammatory, dead, and macrophages). The model was then used to segment all WSIs in the detailed study cohort at the highest 20× resolution. Subsequently, the *cellseg_gsontools*^17^ library was used to quantify the interpretable features both on the full WSI and on the BufferMIL attended patches (MIL attention score value in range [0.2,1.0]).

In total, the employed downstream analysis block extracts 69 spatially aware morphological and cell–cell proximity features. The features fall under several biological themes, namely: tumor size, stroma richness (tumor-induced stroma), neoplastic nuclei pleomorphism (irregular shapes and sizes), spatial proximity-based features of neoplastic cells with connective cells and immune cells (immune infiltration/evasion), level of immune cell clustering, localization, and organization (immune activation). Crucially, the feature set includes an in-depth quantification of cell–cell spatial relationships and clustering metrics derived using graph-based neighborhood analysis. Specifically, the analysis focuses on the interaction of neoplastic cells with three key microenvironmental components (connective/stromal cells, inflammatory cells, and macrophages) within defined spatial bandwidths (100–128 pixels). These metrics quantitatively capture immune infiltration and the localization/organization of immune activation by calculating local cell-type counts, fractions, and diversity (Simpson index) within each cell's neighborhood.

#### Classification block

The derived 69 features are all interpretable by human experts; nonetheless, it would be impractical for a pathologist to manually review all of them to identify the most relevant features associated with the PFI response. To address this challenge, a classification module was developed to focus on interpreting the most significant features, particularly those with the greatest impact on distinguishing between *short/long PFI*. A diverse set of SOTA machine learning classifiers is employed, including K-nearest neighbors, support vector machines (with both linear and RBF kernels), Gaussian process classifier, decision trees, random forest, multi-layer perceptron, AdaBoost, Gaussian Naive Bayes, XGBoost, LightGBM, logistic regression, bagging, histogram-based gradient boosting, and TabPFN. Each model was evaluated through 3-fold cross-validation using a fixed random state (42), and classifiers achieving strong performance (AUC and F1-score >0.8) were retained for downstream analysis. To ensure robust and model-agnostic feature evaluation, feature importance scores from the best-performing classifiers are aggregated. This aggregation was complemented by the use of two independent interpretability methods: SHapley Additive exPlanations (SHAP)^28^ and Local Interpretable Model-agnostic Explanations (LIME).^27^ SHAP quantifies the contribution of each feature based on co-operative game theory, whereas LIME approximates the model's decision boundary by locally fitting interpretable surrogate models. For each fold and each model, feature rankings are computed using SHAP, LIME, and the model's native importance metric (when applicable). Rather than applying an arbitrary cutoff, a progressive classification strategy was adopted. Features were incrementally added to the classifiers according to the aggregated importance ranking (highest feature importance, mean of the model's native importance metric, SHAP and LIME, and lowest RRA score), and performance was re-evaluated at each step. This allowed us to identify the “elbow point”—the point beyond which additional features no longer improved predictive performance significantly or led to overfitting. The number of features corresponding to this inflection point was retained as the optimal subset. In the experiments, this strategy resulted in the selection of the top seven features, which demonstrated consistent and strong predictive value for PFI classification.

## Results

PATHOS consists of three modules. The first module, the *MIL block*, employs a MIL architecture with attention mechanisms to identify informative regions within the WSIs strongly associated with treatment response. The second module, the *segmentation and downstream analysis block*, leverages panoptic segmentation to extract detailed features from these attention-weighted regions, quantitatively representing relevant morphological characteristics. Finally, the third module, the *classification block*, utilizes a classification model to predict treatment response based on the extracted features while identifying the most discriminative features for pathologists.

### MIL block classification performance

The 176 omental WSIs are employed to evaluate the performance of the MIL block using a 5-fold cross-validation strategy. BufferMIL model performances are reported in Table 1 and compared to SOTA methods DSMIL and GDSMIL (configuration parameters are reported in Appendix Table A.2).

**Table 1:** Comparison of the performance for BufferMIL, DSMIL and GSMIL. AUC measures the ability to distinguish between the two classes: accuracy reflects the proportion of correctly classified WSIs; F1-score provides a harmonic mean of precision and recall, and is calculated for both *short* and *long PFI* classes. Results are based on the average performance across a 5-fold cross-validation on all WSIs available.

Model	AUC	Accuracy	F1-score *Short PFI*
BufferMIL	0.93 ± 0.19	0.89 ± 0.25	0.90 ± 0.24
DSMIL	0.88 ± 0.21	0.77 ± 0.13	0.78 ± 0.31
GDSMIL	0.75 ± 0.15	0.67 ± 0.08	0.62 ± 0.26

The BufferMIL model outperformed the GDSMIL and DSMIL models on both the *short* and *long PFI*, achieving an overall accuracy of 89% and an AUC of 93%.

**Fig. 2:**
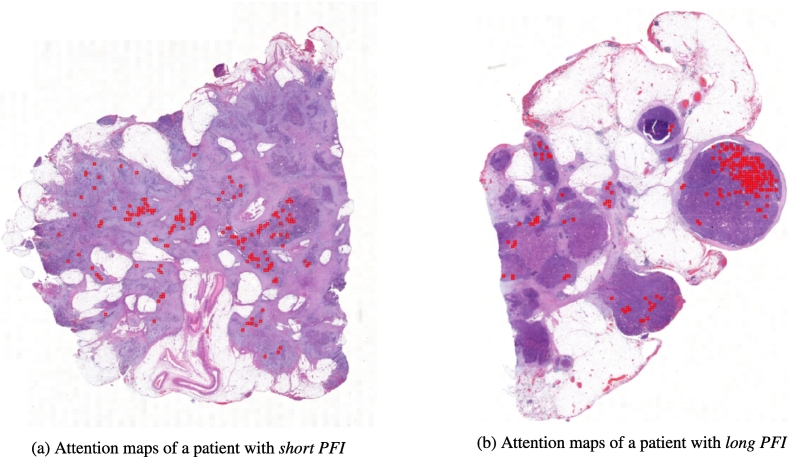
Example of attention maps obtained as output of the MIL block: the high attention patches are visualized in red in the figure. (For interpretation of the references to color in this figure legend, the reader is referred to the web version of this article.)

Because BufferMIL drives the prediction by exploiting a buffer of patches instead of the most significant one, it may offer a more comprehensive view of the ovarian cancer heterogeneity. Additionally, the BufferMIL model outputs an attention map for each WSI, which can be visualized to gain insights into the model's decision-making process. To assess the explainability of the PATHOS framework, Fig. 2 provides an attention map example obtained with the MIL module, to be further processed using the downstream analysis block.

The attention maps in Fig. 2 showed that only a small, localized area within the WSI (reported in red color) was important for predicting *short* and *long PFI*. Specifically, the high attention patches made up 9.77% ± 0.44 of all patches, suggesting that the discriminative signal for the model is localized to a small region.

### PATHOS explainable feature analysis

The classification performance of all models is reported in Table 2. Despite their architectural diversity—ranging from linear models to ensemble methods and neural networks—all classifiers demonstrated consistently high performance in predicting PFI, highlighting the strong predictive value of the extracted features. This robustness across heterogeneous models underscores the relevance and generalizability of the features, irrespective of the underlying learning paradigm.

**Table 2:** Comparison of classification performance using the 69 features from high-attention patches (results highlighted with °) vs entire WSIs (results highlighted with •). Metrics reported as mean ± standard deviation over cross-validation folds. F1-score is reported for short PFI only.

Model	Configuration	AUC	Accuracy	F1-score
AdaBoost	°	0.97 ± 0.02	0.90 ± 0.02	0.90 ± 0.04
	•	0.61 ± 0.15	0.64 ± 0.07	0.71 ± 0.07
Bagging	°	0.95 ± 0.03	0.90 ± 0.04	0.91 ± 0.04
	•	0.56 ± 0.14	0.53 ± 0.06	0.66 ± 0.06
Decision tree	°	0.89 ± 0.03	0.89 ± 0.03	0.90 ± 0.02
	•	0.56 ± 0.11	0.57 ± 0.05	0.66 ± 0.06
Gaussian process	°	0.95 ± 0.05	0.89 ± 0.08	0.89 ± 0.09
	•	0.61 ± 0.12	0.55 ± 0.08	0.67 ± 0.07
HistGradientBoosting	°	0.97 ± 0.03	0.91 ± 0.04	0.91 ± 0.06
	•	0.66 ± 0.17	0.63 ± 0.10	0.71 ± 0.07
LightGBM	°	0.97 ± 0.02	0.89 ± 0.06	0.89 ± 0.05
	•	0.63 ± 0.20	0.62 ± 0.14	0.69 ± 0.12
Linear SVM	°	0.90 ± 0.09	0.91 ± 0.07	0.90 ± 0.09
	•	0.62 ± 0.08	0.59 ± 0.04	0.71 ± 0.04
Logistic regression	°	0.88 ± 0.07	0.91 ± 0.05	0.91 ± 0.08
	•	0.63 ± 0.11	0.63 ± 0.05	0.70 ± 0.07
Naive Bayes	°	0.88 ± 0.07	0.79 ± 0.10	0.82 ± 0.10
	•	0.55 ± 0.14	0.51 ± 0.12	0.46 ± 0.12
Nearest neighbors	°	0.93 ± 0.06	0.89 ± 0.06	0.89 ± 0.09
	•	0.56 ± 0.17	0.54 ± 0.12	0.60 ± 0.14
Neural net	°	0.92 ± 0.09	0.90 ± 0.06	0.90 ± 0.08
	•	0.62 ± 0.08	0.62 ± 0.06	0.67 ± 0.08
RBF SVM	°	0.90 ± 0.07	0.72 ± 0.08	0.71 ± 0.08
	•	0.45 ± 0.09	0.60 ± 0.11	0.75 ± 0.09
Random forest	°	0.96 ± 0.04	0.86 ± 0.05	0.86 ± 0.07
	•	0.64 ± 0.16	0.61 ± 0.05	0.72 ± 0.05
TabPFN	°	0.97 ± 0.02	0.89 ± 0.03	0.89 ± 0.05
	•	0.61 ± 0.20	0.66 ± 0.13	0.73 ± 0.10
XGBoost	°	0.98 ± 0.02	0.92 ± 0.04	0.92 ± 0.05
	•	0.61 ± 0.16	0.63 ± 0.10	0.71 ± 0.09

To construct a model–agnostic feature importance ranking, results from all classifiers that achieved both AUC and F1-score greater than 0.8 for both *short* and *long PFI* classes were integrated. The only model excluded from this aggregation was the RBF SVM, which failed to meet the F1-score threshold for either class. For comparison purposes, the classification performance obtained when using features extracted from the entire WSI for each model is also reported in Table 2. The parameters employed for each tested model are provided in Appendix Table A.3.

Across all models, a substantial drop in performance is observed when using WSI-level features instead of attention-guided patch-level features. This decline affects all metrics—particularly F1-score and AUC—highlighting the advantage of focusing on the most informative regions rather than aggregating information from the entire slide indiscriminately.

To further assess the link between PATHOS framework predictions and overall survival (OS) and to explore PATHOS generalizability on OS, a Kaplan–Meier survival analysis was conducted, using death as the outcome and OS duration as the time variable.

**Fig. 3:**
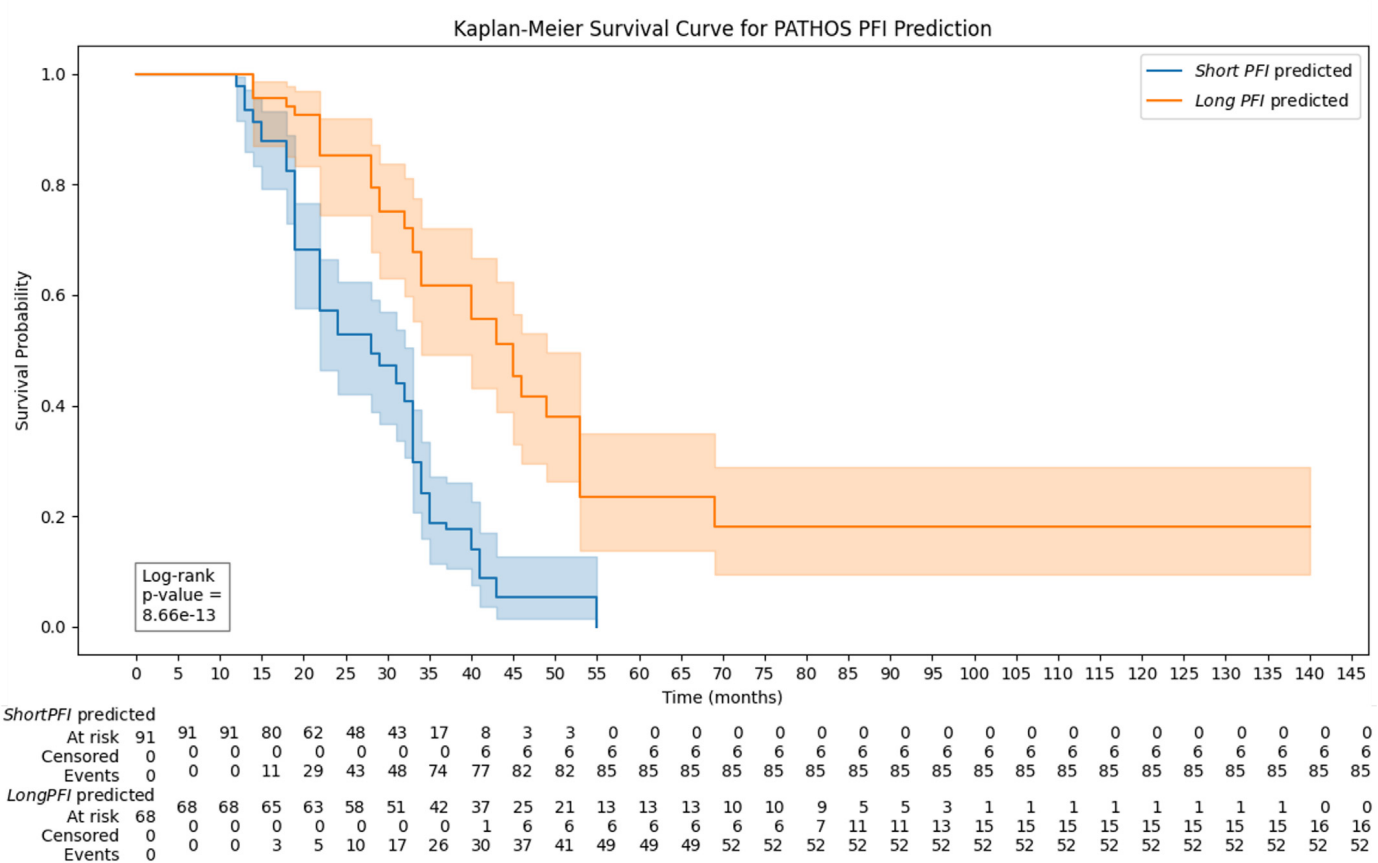
Kaplan–Meier plot showing survival probability over time for patients predicted as *short PFI* or *long PFI.*

As shown in Fig. 3, the PATHOS framework predictions exhibited significant discriminative ability for OS within the studied cohort. This result is consistent with expectations, as PFI and OS prediction are distinct clinical endpoints; however, they are inherently related, both reflecting patient prognosis and response to therapy.

**Fig. 4:**
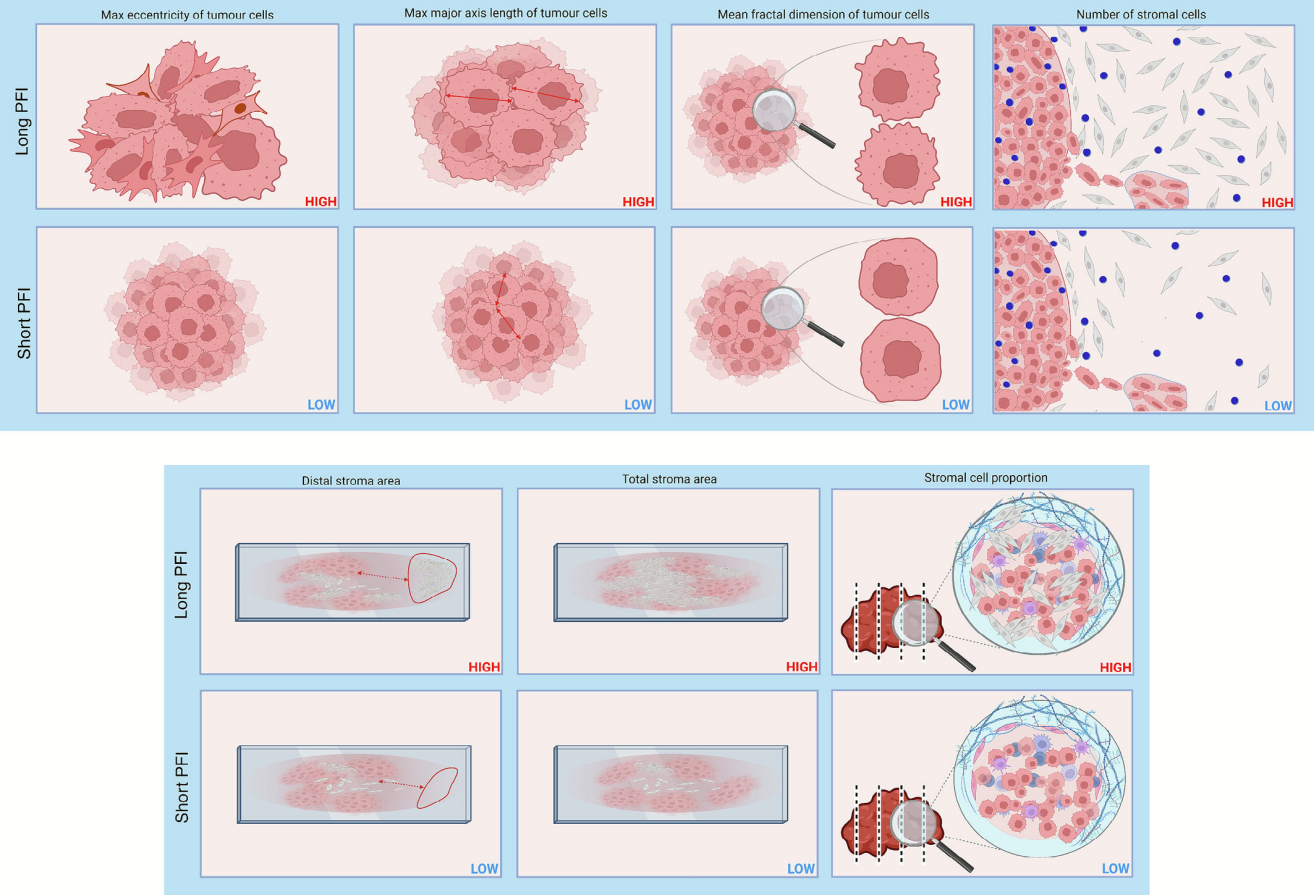
Pictorial illustration of the seven top-ranked morphological features identified by the PATHOS framework. For each feature, representative examples are shown for both long PFI (top row) and short PFI (bottom row) classes. These visual representations highlight the phenotypic differences underpinning each predictive feature across the two prognostic groups. These images are illustrative examples meant to convey the visual phenotype associated with each feature, not a formal definition or exact computation. Quantitative feature values were primarily measured on model-identified high-attention regions; for comparison, the same features were also extracted from whole slides to evaluate differences in classification performance. The images shown are representative of the high-attention regions and are provided for visual guidance only.

Following the cross-validation process and model–agnostic feature importance ranking step, the PATHOS framework returned a final list of seven predictive features reported in Table 3. In Fig. 4, pictorial representations are provided to visually illustrate these features, including examples for both short PFI and long PFI classes.

**Table 3:** Summary of selected histopathological features. Features are associated with treatment response and distinguish between short and long PFI in ovarian HGSC patients.

Feature	Description
Max tumor cell eccentricity	Captures the highest degree of elongation among tumor cells, reflecting morphological irregularity.
Max tumor cell major axis length	Represents the maximum length of tumor cells, which may reflect mesenchymal-like morphology.
Mean tumor cell fractal dimension	Quantifies the average structural complexity of tumor cell shapes, with higher values denoting more irregular boundaries.
Number of stromal cells	Total count of stromal cells, representing the abundance of stromal cells in the tissue.
Distal stromal area	The stromal area located away from the tumor–stroma interface, describing the spatial extent of non-adjacent stroma.
Total stromal area	The entire area occupied by stromal tissue in the analyzed tissue section, capturing overall stromal content.
Stromal cell proportion	Ratio of stromal cells to the total number of cells, indicating the relative dominance of the stromal component in the area.

**Fig. 5:**
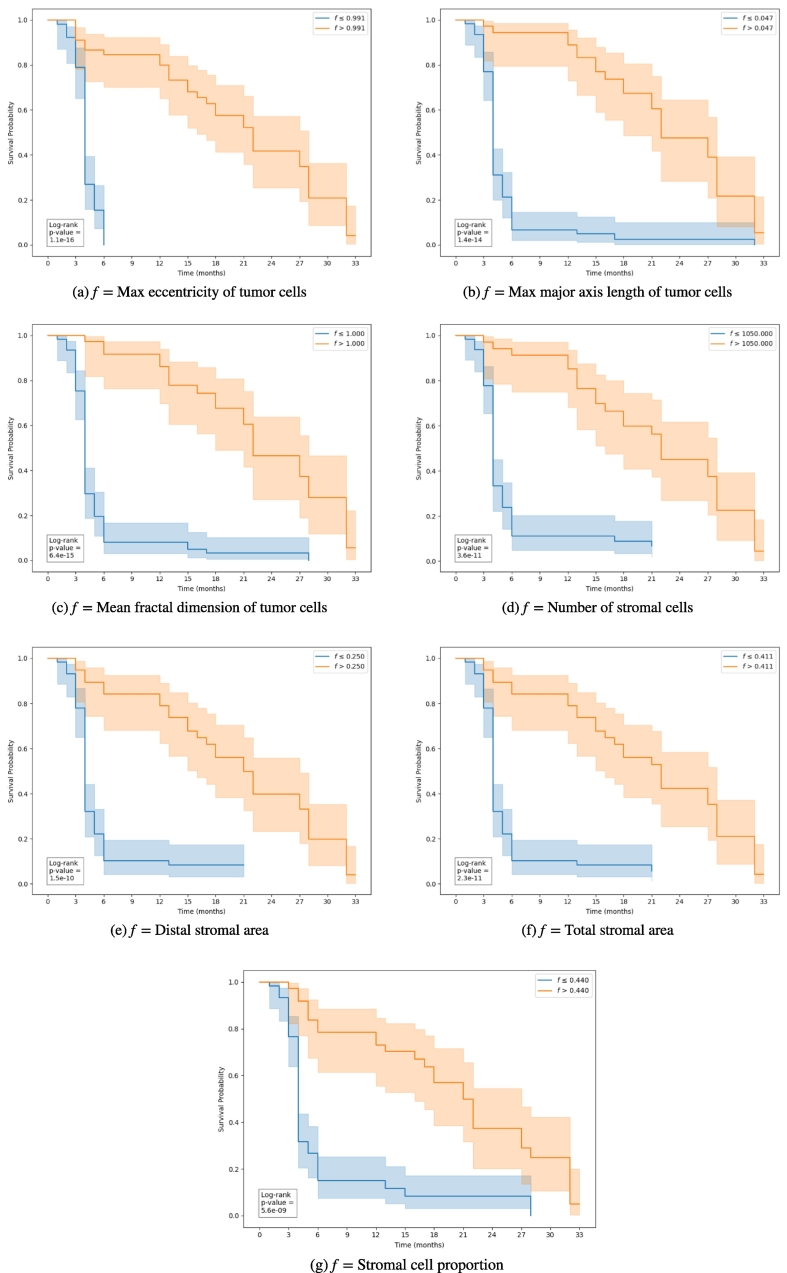
Kaplan–Meier plots depicting survival probability over time for the seven most informative features used in the PFI prediction task.

Additionally, the seven features were investigated through Kaplan–Meier survival analysis (Fig. 5), with the reinstatement of therapy as the event of interest and PFI (expressed in months for readability) as the time variable. Notably, all seven features were identified within this dataset as significantly discriminative for PFI.

## Discussion

The MIL block plays a crucial role in the PATHOS framework, as it generates attention maps that identify and concentrate on the most relevant regions within the WSI for the prediction of treatment response in NACT patients. This method offers two primary benefits. From a semantic perspective, it filters out irrelevant image patches, highlighting only the most significant regions and improving the overall interpretability of the results. Moreover, the MIL block selected, on average, only 9.769% ± 0.438 of patches per WSI as relevant for the prediction, substantially reducing the data volume (hence the computational load) without compromising, and indeed enhancing the overall model performance. This highlights the critical role of the MIL block in balancing computational efficiency and predictive accuracy.

Prior work on spatial heterogeneity in the tumor microenvironment (TME) has employed statistical approaches such as Getis–Ord hotspot analysis and spatial heatmaps to characterize intratumoral clustering of immune infiltrates, establishing that stromal and immune cell organization carries prognostic significance.^26^ In contrast, PATHOS employs a novel integration of attention-guided MIL with panoptic segmentation to automatically identify morphologically and microenvironmentally informative regions. By providing both computational efficiency through selective patch analysis and interpretability through attention maps, PATHOS reveals that fine-grained spatial characteristics of the TME, not simply global cell counts, are key determinants of PFI. Cancer heterogeneity, driven by molecular and microenvironmental differences, including genetic, epigenetic, and transcriptomic diversity, is emphasized in recent reviews as a critical determinant of patient outcome that requires personalized treatment approaches.^21^ In this context, multimodal frameworks that integrate histopathology with molecular data, such as OXA-MISS,^23^ have highlighted the potential of combining complementary sources of information. PATHOS contributes by providing an explainable AI framework that visually identifies and quantifies the histopathological manifestations of this heterogeneity within individual tumors, effectively complementing molecular diagnostics (e.g., next-generation sequencing) and enabling H&E image-based stratification of treatment response.

The effectiveness of the presented approach was evaluated by comparing the classification performance using the seven identified features in Table 3 computed on the most attended patches versus the same features computed over the entire WSI. Table 4 reports the confusion matrices and classification metrics obtained by applying the best-performing models in each setting: XGBoost trained on attention-based patches, and HistGradientBoosting trained on features extracted from the whole-slide. As highlighted in this table, selecting only the most important features is not sufficient; attention-guided region selection is crucial for achieving high prediction performance. Across all evaluations, models consistently show superior performance when features were extracted from attention-based patches. Specifically, XGBoost achieved an accuracy of 92% and AUC of 98% on attention-based inputs, compared to only 60% accuracy and 58% AUC on full-slide features. Even HistGradientBoosting, the best performer on whole-slide data, improved substantially when applied to filtered patches (AUC: 99% vs 61%). These findings confirm that focusing on the most informative regions identified by the MIL attention mechanism leads to more accurate predictions, particularly enhancing the classification of the challenging *short PFI* cases.

**Table 4:** Classification results comparing the best-performing models across feature configurations. Subfigures (a) and (b) show the performance of the best model trained on whole-slide features, whereas (c) and (d) show the performance of the best model trained on attention-guided features. In both cases, the models were evaluated using only the seven features identified as most relevant by the importance analyses, rather than all available features. Applying the model to the attentioned patches improves classification accuracy, confirming the value of attention-based region selection.

	Predicted *Long*	Predicted *Short*
*(a) HistGradientBoosting on entire WSI features*		
Actua		
*Long*	42.5%	57.5%
Actual		
*Short*	25.8%	74.2%
	Accuracy	AUC
	0.65	0.61
*(b) HistGradientBoosting on high attention patches*		
Actual		
*Long*	94.3%	5.7%
Actual		
*Short*	10.1%	89.9%
	Accuracy	AUC
	0.92	0.99
*(c) XGBoost on entire WSI features*		
Actual		
*Long*	36.8%	63.2%
Actual		
*Short*	29.2%	70.8%
	Accuracy	AUC
	0.60	0.58
*(d) XGBoost on high attention patches*		
Actual		
*Long*	91.2%	8.8%
Actual		
*Short*	7.8%	92.2%
	Accuracy	AUC
	0.92	0.98

Recent machine-learning approaches have successfully demonstrated that immunotyping of the TME reveals distinct molecular subtypes with implications for personalized immunotherapy.^32^ PATHOS provides an orthogonal, histopathology-based approach to characterize TME heterogeneity and predict treatment response using attention mechanisms on standard WSI. It complements molecular work by translating morphological and stromal features visible in histopathology into quantitative image-based biomarkers, potentially enabling similar stratification strategies directly from routinely available WSI and thus, reducing the reliance on complex bulk or single-cell molecular profiling.

To further assess the effect of attention-based patch selection on feature relevance, the incremental feature analysis was replicated using the models trained on features computed from the entire WSI, bypassing the MIL block. Differently from the MIL-based analysis, where only models with both F1 and AUC above 0.8 were considered, in this case, a lower threshold of 0.6 was adopted for both metrics, as none of the classifiers trained on whole-slide features exceeded the stricter criteria. The resulting feature rankings reveal a substantial difference between the two approaches. When attention-based filtering was applied, the most informative features included the maximum eccentricity and maximum major axis length of tumor cells, as well as their mean fractal dimension, highlighting the morphological characteristics of neoplastic cells after NACT. In contrast, using features extracted from the whole-slide, the top-ranked features were the proportion of immune cells, the distal stromal area, and the tumor–stroma interface (TSI) area, which reflect more global aspects of the TME. Some few features, such as the distal stromal area and fractal dimension of tumor cells, were identified as important in both settings, confirming their robustness. However, the MIL-based filtering approach led to a shift in focus toward more localized and discriminative descriptors of tumor morphology and organization. This refinement was particularly beneficial for identifying short PFI cases, reducing false positives and false negatives, and increasing overall performance, raising AUC from 66% to 92%. These results emphasize that integrating MIL-driven region selection not only improves classification accuracy but also enhances the biological relevance and interpretability of the selected features.

The panoptic segmentation and downstream analysis component serves a critical function within the pipeline, as it provides users with a detailed set of human-interpretable features. Whereas the MIL block generates attention maps that visually highlight the most relevant regions, these maps alone do not provide a comprehensive list of interpretable features. The downstream analysis block complements the MIL component by extracting biologically and clinically meaningful features from the WSI. The classification block further enhances the pipeline by conducting a feature importance analysis, effectively performing feature selection. This process distils the extensive set of features generated by the panoptic segmentation and downstream analysis into a more manageable subset, enabling to focus on and interpret the most salient features.

The analysis revealed distinct patterns in the TME associated with *long* versus *short PFI* in ovarian HGSC. All top-ranked features extracted from the most informative tissue regions, identified through attention-based filtering, were found to have generally higher values in *long PFI* cases. These include a higher eccentricity and major axis length of tumor cells, increased tumor cell fractal complexity, a larger stromal area and number of stromal cells, and greater distal stroma coverage. Together, these findings suggest that long PFI cases are characterized by increased tumor cell pleomorphism and higher stromal content following NACT, potentially reflecting distinct tissue remodelling patterns associated with treatment response. Notably, increased fibroinflammatory stromal content has previously been associated with favorable treatment outcome and is a key component of the chemotherapy response score (CRS), which is widely used in routine pathology.^4^^,^^8^ However, CRS does not incorporate post-NACT morphological features of tumor cells. Our findings suggest that such features, quantified here through interpretable computational analysis, may complement existing CRS criteria and offer additional value for clinical evaluation of treatment response. Additionally, unifying all blocks into a single framework leads to a reciprocal enhancement of their respective strengths, demonstrating the utility, purpose, and added value of PATHOS integration.

The histopathological features identified by PATHOS, such as tumor cell morphology and stromal architecture, may reflect underlying molecular heterogeneity in response to NACT. Although this study focuses on image-based prediction, the attention-guided regions highlighted by PATHOS are spatially discrete and could be suitable for laser capture microdissection. Future work integrating targeted microdissection or spatial transcriptomics may validate the biological relevance of these features, through both *intralesional* comparisons (between distinct regions within the same tumor) and *interlesional* comparisons (across tumors with similar histopathological traits), further enhancing the interpretability and biological basis in response to NACT of the framework.

## Conclusions

This study demonstrated the efficacy of the PATHOS framework in the explainability of treatment response after NACT in ovarian HGSC using H&E WSIs. The integration of attention-based MIL, panoptic segmentation, and feature-based classification enabled the identification of critical histopathological features, improving prediction accuracy while maintaining computational efficiency. The sequential process of the three blocks demonstrated the true value of the framework, where each module complements the others to achieve superior results compared to their independent use.

By leveraging attention maps, the framework effectively isolated task-relevant regions, reducing data volume without compromising accuracy. This confirms the benefit of focusing on the most informative regions, leading to more reliable predictions. The extracted features revealed biologically significant insights, particularly in distinguishing short and long PFI cases, highlighting tumor cell morphology and stromal content as key determinants.

The PATHOS framework presents a significant step forward in personalized treatment strategies, offering a robust, interpretable, and efficient tool for explainable models in ovarian HGSC management. External validation on larger, diverse cohorts is essential to confirm generalizability. Future integration with omics data may further enhance predictive performance.

## Declaration of generative AI and AI-assisted technologies in the writing process

During the preparation of this work, the authors utilized ChatGPT and Gemini to enhance the manuscript's readability. After using these tools, the authors reviewed and edited the content as needed and take full responsibility for the content of the published article.

## Declaration of competing interest

The authors declare the following financial interests/personal relationships which may be considered as potential competing interests:

**Table:** 

Elisa Ficarra reports financial support was provided by Horizon 2020 - the Framework Programme for Research and Innovation. Marta Lovino reports administrative support, article publishing charges, and equipment, drugs, or supplies were provided by University of Modena and Reggio Emilia Department of Engineering Enzo Ferrari. Marta Lovino reports financial support was provided by Horizon 2020 - the Framework Programme for Research and Innovation. Miccolis Francesca reports financial support was provided by Horizon 2020 - the Framework Programme for Research and Innovation. Francesca Miccolis reports administrative support, article publishing charges, and equipment, drugs, or supplies were provided by University of Modena and Reggio Emilia Department of Engineering Enzo Ferrari. Oskari Lehtonen reports financial support was provided by Horizon 2020 - the Framework Programme for Research and Innovation. Sampsa Hautaniem reports financial support was provided by Horizon 2020 - the Framework Programme for Research and Innovation. Anni Virtanen reports financial support was provided by Horizon 2020 - the Framework Programme for Research and Innovation. Elisa Ficarra reports administrative support, article publishing charges, and equipment, drugs, or supplies were provided by University of Modena and Reggio Emilia Department of Engineering Enzo Ferrari. If there are other authors, they declare that they have no known competing financial interests or personal relationships that could have appeared to influence the work reported in this article.

## Figures and Tables

**Table A.1:** Parameters for panoptic segmentation with Cellpose.

Parameter	Value	Description
Normalization	minmax	Normalization method
Activation functions	softmax	Activations for segmentation
Stride	180	Stride used for tiling
Padding	20	Padding for segmentation
Model	“cellpose”	Base model used for segmentation
Model type	“plus”	Variant of the Cellpose model
Number of types (ntypes)	7	Number of object types
Number of tissues (ntissues)	7	Number of tissue types
Encoder name	“tf_efficientnetv2_l”	Backbone encoder
Encoder pretrained	False	Use of pretrained weights
Depth	5	Depth of the model
Output channels	(256, 256, 128, 64, 32)	Channels in the output layers
Layer depths	(4, 4, 4, 4, 4)	Depths of each layer in the model

**Table A.2:** General and BufferMIL-specific parameters used in the experiments.

Parameter	All models	BufferMIL-specific
Dropout rate	0.2	–
Learning rate	0.0002	–
Weight decay	0.005	–
Number of epochs	200	–
Seed	48	–
Buffer aggregation	–	Mean
Number of buffer patches (ntop)	–	3
Buffer update frequency	–	10

**Table A.3:** Classifiers and their parameters used in the evaluation.

Classifier	Parameters
AdaBoost	random_state = 42
Bagging	estimator = DecisionTreeClassifier(), random_state = 42
Decision tree	max_depth = 5, random_state = 42
Gaussian process	kernel = 1.0 * RBF(1.0), random_state = 42
HistGradient-boosting	random_state = 42
K-neighbors	n_neighbors = 3
LightGBM	random_state = 42
Linear SVM	kernel = “linear”, C = 0.025, probability = True, random_state = 42
Logistic regression	penalty = ‘l2’, solver = ‘liblinear’, random_state = 42
MLP (neural network)	alpha = 1, max_iter = 1000, random_state = 42
Naive Bayes	(Default Parameters)
Quadratic discriminant analysis	(Default Parameters)
RBF SVM	kernel = “rbf”, gamma = 2, C = 1, probability = True, random_state = 42
Random forest	max_depth = 5, n_estimators = 10, max_features = 1, random_state = 42
TabPFN	(Default Parameters)
XGBoost	use_label_encoder = False, eval_metric = ‘logloss’, random_state = 42

## Data Availability

The datasets used in this study are not publicly available due to legal and ethical restrictions.

## Appendix A Appendix

### A.1 Panoptic segmentation model

Our panoptic segmentation model, based on the Cellpose model with an extra tissue segmentation branch is a multi-task U-Net CNN with a shared backbone encoder and task-specific decoder branches for tissue, and nuclei segmentation.^2^^,^^3^ An ImageNet pretrained EfficientNetV2-L was selected as the encoder backbone due to the high performance of the architecture in image classification tasks.^4^^,^^5^

### A.2 Panoptic segmentation training data

We collected and semi-manually annotated a training dataset of 197 H&E-stained image crops at 20× magnification of various sizes. For each image crop, five different nuclear types and six different tissue region types were manually annotated by drawing the border of the nucleus and tissue regions with the QuPath software.^1^ The nuclei and tissue types were chosen by a pathologist based on the visual detectability of the distinct classes from the H&E-stained images. The classes for the nuclei were neoplastic, connective, inflammatory, dead, and macrophages, whereas the tissue region types were stroma, omental fat, tumor, necrosis, hemorrhage, and serum. In total, the training dataset contains 98,468 annotated nuclei and over 699,744,885 pixels of annotated tissue regions. The connective nuclei are the most frequent nuclei type in the data with over 46,100 (∼47%) annotations, followed by 22,761 (∼23%) neoplastic, 19,185 (∼19%) inflammatory, 1859 (∼2%) dead, and 4550 (∼5%) annotated macrophages. The most frequent tissue type is the stromal tissue consisting of 28% of the annotated pixels, followed by 29% of tumor, 20% omental fat, 9% hemorrhage, and 13% of necrosis.

### A.3 Panoptic segmentation model training

The image crops in the training data were tiled into 224 × 224 px patches with a sliding window approach. The stride of the sliding window was set to 32 pixels to introduce overlap between patches due to *StrongAugment* data augmentation regimen, which was used to reduce batch effects and domain shifts. The model was trained for 40 epochs with a batch size of 10.

### A.4 Classification block: Feature derivation and final selection process

The final list of features was obtained through a multi-step procedure designed to ensure robustness and interpretability:

1. Data preprocessing: starting from features extracted from attention-based regions within WSIs, missing values were imputed by class-wise mean imputation. Features consisting entirely of missing values in at least one class were removed to maintain data quality.
2. Feature filtering: features with near-zero variance were excluded to remove uninformative variables. Features exhibiting high pairwise correlation (above 0.95) were removed to reduce redundancy and improve stability.
3. Normalization: features were standardized using training data statistics (zero mean, unit variance), with the same transformation applied to test data to ensure comparability.
4. Model training and cross-validation: multiple classifiers were trained using stratified group k-fold cross-validation, generating predictions and probabilities for each fold.
5. Feature importance computation: for each fold and model, feature importance was calculated using three complementary methods:
  • Permutation importance: evaluates the impact on model accuracy by randomly shuffling feature values.
  • LIME (Local Interpretable Model-agnostic Explanations): provides local approximations explaining individual predictions.
  • SHAP (SHapley Additive exPlanations): assigns contribution scores to features based on co-operative game theory principles.
6. Aggregation of importance scores: importance scores from permutation, LIME, and SHAP were averaged to produce a combined importance value per feature, per fold, and per model.
7. Cross-model aggregation and ranking: feature importance summaries from all folds and models were merged. Features missing in some models were assigned zero importance for uniform comparison. Aggregate statistics are computed for each feature, including mean importance, standard deviation, *Z*-score,^2^ percentage of total importance, and robust rank aggregation^3^ (RRA).
8. Aggregated feature ranking: to create a single, definitive ordering of all features, we aggregated the metrics derived from the cross-model ranking (Z-score, mean importance, and RRA score). This process resulted in a ranked list of features, sorted by optimizing the three criteria simultaneously: maximizing the mean importance score (including SHAP and LIME), maximizing the Z-score, and minimizing the RRA score (indicating consistent high ranking across models).
9. Optimal feature subset selection (elbow point): the final determination of the optimal feature set size was achieved through *Incremental Feature Analysis*. Using the aggregated feature ranking derived in step 8, we iteratively added features one by one to the input of all retaining classifiers (starting from *k* = 1). At each step, predictive performance metrics (AUC, accuracy, and F1-score) were computed and averaged across all folds and models. The process concluded at the point (the “elbow point”), where the marginal increase in predictive performance achieved a plateau, signifying that further features did not contribute significantly to the predictive power while mitigating the risk of overfitting.

This pipeline ensures that the final feature set reflects consensus across multiple models and folds, providing a robust and interpretable basis for subsequent predictive modeling.

## References

[1] Bankhead P., Loughrey M., Fernández J. (2017). Qupath: open source software for digital pathology image analysis. Sci Rep.

[2] Bontempo G., Bartolini N., Lovino M., Bolelli F., Virtanen A., Ficarra E. (2023). International Conference on Image Analysis and Processing.

[3] Bontempo G., Lumetti L., Porrello A., Bolelli F., Calderara S., Ficarra E., Foresti G.L., Fusiello A., Hancock E. (2023). Image Analysis and Processing.

[4] Böhm S., Faruqi A., I., S., M., L., E., B., A., J., A., F., D., E., T., D., JL., S., LS., C., AV., T., ND., L., CB., G., N., S (2015). Chemotherapy response score: development and validation of a system to quantify histopathologic response to neoadjuvant chemotherapy in tubo-ovarian high-grade serous carcinoma. J Clin Oncol.

[5] Carbonneau M., Cheplygina V., Granger E., Gagnon G. (2018). Multiple instance learning: a survey of problem characteristics and applications. Pattern Recogn.

[6] Caron M., Touvron H., Misra I. (2021). Emerging Properties in Self-Supervised Vision Transformers. https://arxiv.org/abs/2104.14294.

[7] Chuang W., Chang S., Yu W. (2020). Successful identification of nasopharyngeal carcinoma in nasopharyngeal biopsies using deep learning. Cancers.

[8] Cohen P., Powell A., Böhm S. (2019). Pathological chemotherapy response score is prognostic in tubo-ovarian high-grade serous carcinoma: a systematic review and meta-analysis of individual patient data. Gynecol Oncol.

[9] Etzerodt A., Moulin M., Doktor T.K. (2020). Tissue-resident macrophages in omentum promote metastatic spread of ovarian cancer. J Exp Med.

[10] Friedlander M., Trimble E., Tinker A. (2011). Clinical trials in recurrent ovarian cancer. Int J Gynecol Cancer Off J Int Gynecol Cancer Soc.

[11] Iwagoi Y., Motohara T., Hwang S., Fujimoto K., Ikeda T., Katabuchi H. (2021). Omental metastasis as a predictive risk factor for unfavorable prognosis in patients with stage iii–iv epithelial ovarian cancer. Int J Clin Oncol.

[12] Jenkinson E., Arandjelović O. (2024). Whole slide image understanding in pathology: what is the salient scale of analysis?. BioMedInformatics.

[13] Ji R., Yong L., He C., Zhu X., He A., Lu Y. (2020). Detection and analysis of multiple biomarkers in ovarian cancer: clinical significance in diagnosis, treatment, and prognosis evaluation. Gland Surg.

[14] Kannan S., Morgan L., Liang B. (2019). Segmentation of glomeruli within trichrome images using deep learning. Kidney Int Rep.

[15] Kim B., Park Y., Kim B. (2018). Diagnostic performance of ca 125, he4, and risk of ovarian malignancy algorithm for ovarian cancer. J Clin Lab Anal.

[16] Le Saux O., Ray-Coquard I., Labidi-Galy S.I. (2021). Challenges for immunotherapy for the treatment of platinum resistant ovarian cancer. Semin Cancer Biol.

[17] Lehtonen O. (2023). https://github.com/okunator/cellseg_gsontools.

[18] Li B., Li Y., Eliceiri K.W. (2020). CoRR abs/2011.08939.

[19] Lin Y., Wang H., Dong L., Shen J. (2023). Federated learning with hyper-network—a case study on whole slide image analysis. Sci Rep.

[20] Lu M.Y., Chen R.J., Wang J., Dillon D., Mahmood F. (2019). Semi-Supervised Histology Classification Using Deep Multiple Instance Learning and Contrastive Predictive Coding. https://arxiv.org/abs/1910.10825.

[21] MacDonald W.J., Purcell C., Pinho-Schwermann M., Stubbs N.M., Srinivasan P.R., El-Deiry W.S. (2025). Heterogeneity in cancer. Cancers.

[22] Mamoor S. (2021).

[23] Miccolis F., Marinelli F., Pipoli V. (2025). MICCAI Workshop on Computational Pathology with Multimodal Data (COMPAYL).

[24] Mohammadi M., Cooper J., Arandelović O. (2022). Weakly supervised learning and interpretability for endometrial whole slide image diagnosis. Exp Biol Med.

[25] Myronenko A., Xu Z., Yang D., Roth H.R., Xu D. (2021). Medical Image Computing and Computer-Assisted Intervention.

[26] Nearchou I.P., Christodoulou C.D.C.S., Kaimakis M.L.L. (2021). A comparison of methods for studying the tumor microenvironment’s spatial heterogeneity in digital pathology specimens. J Pathol Inform.

[27] Ribeiro M.T., Singh S., Guestrin C. (2016). Proceedings of the 22nd ACM SIGKDD International Conference on Knowledge Discovery and Data Mining, San Francisco, CA, USA, August 13-17, 2016.

[28] Scott M., Su-In L. (2017). A Unified Approach to Interpreting Model Predictions. https://proceedings.neurips.cc/paper_files/paper/2017/file/8a20a8621978632d76c43dfd28b67767-Paper.pdf.

[29] Stringer C., Wang T., Michaelos M. (2021). Cellpose: a generalist algorithm for cellular segmentation. Nat Methods.

[30] Tizhoosh H., Pantanowitz L. (2018). Artificial intelligence and digital pathology: challenges and opportunities. J Pathol Inform.

[31] Wang J., Mao Y., Guan N., Xue C.J. (2024). Advances in Multiple Instance Learning for Whole Slide Image Analysis: Techniques, Challenges, and Future Directions. https://arxiv.org/abs/2408.09476.

[32] Zeng D., Li Y., Lv H. (2025). Immunotyping the tumor microenvironment reveals molecular heterogeneity for personalized immunotherapy in cancer. Adv Sci (Weinheim, Baden-Wurttemberg, Germany).

